# Best practice for the nuclear medicine technologist in CT-based attenuation correction and calcium score for nuclear cardiology

**DOI:** 10.1186/s41824-020-00080-0

**Published:** 2020-07-06

**Authors:** Luca Camoni, Andrea Santos, Marieclaire Attard, Marius Ovidiu Mada, Agata Karolina Pietrzak, Sonja Rac, Sebastijan Rep, Christelle Terwinghe, Pedro Fragoso Costa, on behalf of the Technologist Committee of the European Association of Nuclear Medicine (EANM)

**Affiliations:** 1 Nuclear Medicine Department, University of Brescia and Spedali Civili di Brescia, Piazzale Spedali Civili 1, 25123 Brescia, Italy; 2 Nuclear Medicine Department, Hospital Cuf Descobertas, Lisbon, Portugal; 3 Department of Nuclear Medicine and Radiology, Isala, Zwolle, Netherlands; 4 Wolfson Brain Imaging Centre, University of Cambrige, Cambridge, United Kingdom; 5 Nuclear Medicine Department, Greater Poland Cancer Centre, Poznan, Poland; 6 Electroradiology Department, Poznan University of Medical Sciences, Poznan, Poland; 7 Nuclear Medicine Department, Clinical Hospital Centre Rijeka, Rijeka, Croatia; 8 Nuclear Medicine Department, University Medical Centre Ljubljana, Ljubljana, Slovenia; 9 Nuclear Medicine Department, University Hospitals Leuven, Leuven, Belgium; 10 Department of Nuclear Medicine, University Hospital Essen, University of Duisburg-Essen, Essen, Germany

**Keywords:** Myocardial perfusion imaging, CT, Attenuation correction, Calcium score, Nuclear medicine technologist

## Abstract

The use of hybrid systems is increasingly growing in Europe and this is progressively important for the final result of diagnostic tests. As an integral part of the hybrid imaging system, computed tomography (CT) plays a crucial role in myocardial perfusion imaging diagnostics. Throughout Europe, a variety of equipment is available and also different university curricula of the nuclear medicine technologist are observed. Hence, the Technologist Committee of the European Association of Nuclear Medicine proposes to identify, through a bibliographic review, the recommendations for best practice in computed tomography applied to attenuation correction and calcium score in myocardial perfusion imaging, which courses in the set of knowledge, skills, and competencies for nuclear medicine technologists. This document aims at providing recommendations for CT acquisition protocols and CT image optimization in nuclear cardiology.

## Introduction

Single photon emission computed tomography combined with computed tomography (SPECT/CT) and positron emission tomography combined with computed tomography (PET/CT) hybrid imaging are well established non-invasive hybrid imaging techniques, commonly recognized as providing diagnostic information of metabolic pathways and anatomy (Even-Sapir et al. 2009). Over the last decades, these modalities have experienced a rapid growth and dissemination in clinical practice (Salvatori et al. 2019).

Due to the emerging role of the CT in hybrid nuclear imaging, the community has to adapt the training curricula of the workforce to provide a sufficient set of competencies to master the new techniques (Beyer et al. 2018; Stegger et al. 2008; Delbeke et al. 2012; Medicine EAoN, Radiology ESo 2012; Bischof Delaloye et al. 2007; Fragoso Costa et al. 2017; Costa et al. 2019)

Nuclear medicine technologists (NMT) training curricula varies among European countries (Matos et al. 2015) endowing the NMT with different practical skills in the field of nuclear cardiology. Additionally, different acquisition protocols are currently used across Europe (Lindner et al. 2016). This has motivated the Technologist Committee of the European Association of Nuclear Medicine (EANM) to publish a consensus document emphasizing the competencies in hybrid imaging aiming to set the standards for NMT education in Europe (Fragoso Costa et al. 2017). It has been demonstrated that the diagnostic performance in myocardial perfusion imaging (MPI) is highly influenced by image acquisition and processing optimization (Dondi et al. 2018), where CT-based attenuation correction (AC) and the coronary artery calcium score (CCS) play a key role in the optimization process of the MPI diagnostic performance. The AC leads to an improvement of the image quality and the CCS has an additive prognostic value (Flotats et al. 2011).

The purpose of this review is to continue the NMT education analysis started by the EANM Technologist Committee, particularly identifying the best practice and technique applied to AC and/or CCS with SPECT or PET MPI. Through a bibliographical review, the NMT competencies are contextualized to the current status of CT in the MPI, focusing on practical methods.

### Hybrid imaging and radiation protection

Hybrid imaging is the fusion of emission (SPECT or PET) and transmission (CT) images. This provides both patient morphological and functional information with stand-alone, side-by-side, or merged interpretation of the data sets.

In hybrid imaging, both emission and transmission data sets contribute to the image information, help to optimize the diagnostic performance, and reduce the number of equivocal findings (Flotats et al. 2011).

Hybrid imaging, combining nuclear medicine techniques with CT has contributed substantially to the development for nuclear medicine imaging, in which it complements the limitations of NMI, particularly at the level of spatial resolution and tissue contrast principles. Notably for PET/CT, the synergetic effect of AC and one-stop-shop concept for many clinical indications renders single modality simply not practicable.

Due to this relevance, the NMT has to be aware of the implications of the CT, when performing hybrid imaging in different clinical applications as these can affect the diagnosis.

Moreover to providing high quality and artifact-free images, the NMT has to be aware of the variety of hardware and software solutions, technical factors, parameters (Seibert 2004; Seibert and Boone 2005), principals of multi-detector CT (Goldman 2008), and artifacts (Popilock et al. 2008; Kalisz et al. 2016) that significantly influence the final CT image quality, while keeping the patient effective dose to an acceptable level. Since hybrid imaging has contributed to an increase in the radiation exposure due to the addition of the CT component to NM, it has become mandatory to consider the clinical purpose of the CT scan. The wide range of radiation exposure associated to the different acquisition protocols and clinical purposes (Iball et al. 2017) has highlighted the need for optimization in hybrid imaging (Salvatori et al. 2019).

Furthermore, the combined use of CT and nuclear medicine technologies calls for additional measures to keep occupational exposures at a reasonable level. All measures to ensure this end, should comply with the requirements for both disciplines. As such, it is fundamental that NMT and radiographers pursue complementary education, which will also increase a layer of knowledge to cover a critical review of the indication principle. Operationally, all exposed workers should adapt their procedures, bearing in mind the determinants of radiation exposure, such as exposure time, distance to source, and proper application of shielding. The use of adequate personal dosimeters and the proper integration in the multidisciplinary team, in particular communication with the medical physics department, facilitates an efficient revision of practices as to keep exposures reasonably low (Massalha et al. 2019).

### CT acquisition parameters and optimization

In this paper, acquisition parameters are considered, and the reconstruction parameters are reported in the AC and CCS sections of this document. The main acquisition parameters are defined as follows:

Tube voltage: represents the electric potential difference (expressed in kilovolts—kV), between the anode and the cathode of the X-ray tube that accelerates the electrons produced by the heated filament cathode towards the anode. Kilovoltage peak—kVp, which is equally to the tube voltage, is the maximum electron energy available from this acceleration. The kilovoltage level strictly determines the radiation penetration of the X-rays. The higher the kilovoltage, the more energetic the X-rays, and the more penetrating these X-rays become.

Tube current: measured in milliamperes (mA), the tube current regulates the amount of X photons that passes through the patient per unit time. However, a more useful measurement is the tube current-time product (mAs), which, as the product of the tube current and the exposure time per rotation (in seconds) gives a measure of the total number of X photons per rotation. In multi-detector CT systems with helical acquisition, the effective tube current-time product is determined by the ratio of tube current-time product to pitch (mAs_eff_).

Tube rotation time: measured in time (seconds), is the period necessary for the tube-detector ensemble to perform a complete rotation around the patient

Collimation: in modern multi-detector CT scanner, the beam collimation is equivalent to the product of the detector configuration (the number of data channels multiplied by the effective detector row thickness)

Table feed: measured in mm/s, represents the movement (mm) of the CT table in one second of time

Pitch: modern hybrid systems employ multi-detector CT (Beyer et al. 2011a; Beyer et al. 2011b). The International Electrotechnical Commission (IEC) has defined the pitch (*P*) of a multi-detector-CT as follows: the ratio between the table motion during one tube rotation (*d*) and the total collimation. The total collimation is equal to the number of slices (*M*) multiplied by the thickness of the collimated slice (*S*). The pitch is given by: *P* = *d*/(*M* · *S*)

Cardiac gating: temporal phase of the R-R interval of the cardiac cycle

Some generic concepts can be considered for the optimization of these parameters in nuclear medicine. The tube voltage typically is based on patient’s size (i.e., body mass), e.g., a wider patient diameter needs a higher voltage to ensure adequate penetration by the X-photons, and it can be set automatically semi-automatically but is most commonly set manually in the acquisition protocol (Yu et al. 2010). For CT contained in the hybrid scanner, the tube voltage is typically between 70 and 140 kV. Lowering the voltage reduces the effective dose and improves image contrast but increases the image noise in the region of interest (ROI). However, the patient effective dose is not determined by the kV alone, being also related to the aforementioned tube current time product, pitch (for helical scans), and the beam-shaping bow-tie filter being used. Furthermore, the effective dose reduction is not proportional, being also affected by other variables like attenuation coefficient of the tissue/substance being scanned. For example, in a CT water phantom, the total absorbed dose was decreased by 28–40% when reducing the tube voltage from 140 to 120 kVp and keeping all the other parameters fixed (Reid et al. 2010; Tamm et al. 2011). Lowering the tube voltage reduces dose only if all other CT parameters are kept constant and the image quality is not defined by automatic tube current modulation (ATCM); otherwise, the system will balance the lower kV by choosing higher milliamperes. Furthermore, a 140-kV tube voltage might be needed to obtain good image quality in overweight patients (Lee et al. 2014; Lee et al. 2018). In nuclear medicine, the CT scanner’s X-ray tube has a wide current range (1–800 mA), depending on the model and generation of the hybrid system, whether they belong to the first generation, where the CT tube was incorporated into the slip ring gantry, or to the most recent systems (Beyer et al. 2011a; Beyer et al. 2011b).

The effective dose is proportional to the current-time product, i.e., when all other factors are kept constant, halving the milliamperes will halve the effective dose. Since it reduces the number of photons, decreasing the milliamperes’ value results in an increase of noise, inversely proportional to the square root of the milliamperes. This increase in noise will also reduce low-contrast visibility (McNitt-Gray 2002). In a CT protocol, the milliamperes can be maintained fixed for all the acquisition volume or can be modulated using the automatic tube current modulation system (ATCM).

The ATCM deserves special attention because it can lead to a significant reduction in the radiation exposure compared to the fixed current approach, when correctly applied (Raman et al. 2013a; Efstathopoulos et al. 2012). At the same time, all manufacturers implement this tool differently and the NMT must take under consideration these differences when the ATCM is applied. The goal of these tools is to maintain the same image quality throughout the whole scan, taking into account both the patient’s size and the characteristic attenuation of each tissue. Through a posteroanterior (PA) localizer radiograph, the ATCM tailors the tube current along the CT longitudinal direction (*z*-axis modulation) decreasing the current for small regions (arms, leg, head, etc.) or for low density organs (lungs).

Additionally, rotational modulation (*xy*-axes modulation) adjusts the tube current according to the projection angle in each rotation of the tube. So, for example, the current through a PA projection is often less than that for a lateral projection because of the smaller amount of tissue to pass through in a PA projection. Some rotational modulation systems require two scout radiographs (PA, mentioned above, and lateral) performed before the CT acquisition. From these two scouts, the software obtains the patient’s size and modulates the tube current during 360° rotation, in order to contrast the attenuation differences along the *x*, *y*, and *z* axes, producing a constant quality of the image and reducing the dose.

In a recent study, it has been shown that not only ATCM features are different among the manufacturers, but also that the localizer setting parameters might be different (orientation, table height, tube voltage). This is true also in different CT models of the same manufacturer. The image quality of the localizer can influence the current adjustment and thus can increase or reduce the patient dose if not used properly (Paolicchi et al. 2020). Each manufacturer has developed specific methods to improve the ATCM system and its influence on image quality.

For the General Electric (GE) CT systems, the ATCM is connected to the minimum-maximum range of the milliampere (to avoid non-diagnostic under- or over-radiation exposure for the patient) and to the level of image noise parameter (GE Healthcare, noise index [NI]). The NI represents the expected level of average image noise in a uniform region. The CT system automatically varies the tube current to maintain the selected NI even if the other parameters are modified. Due to this, the NI becomes a determining factor for the effective dose to the patient. Decreasing the NI by 5%, increases the dose by up to the 10.8%, whereas increasing the NI by 5% decreases the dose by approximately 9.3% of the total effective dose (Kanal et al. 2007).

Philips Healthcare’s CT systems (DoseRight©) have three components: automatic current selection, longitudinal modulation, and angular modulation. The user is able to determine the current tube settings and must define a reference image for scan of a prior patient examination or a pre-stored standard phantom. The software modulates the tube current to the lowest possible levels to achieve the same noise levels as the reference image. The DoseRight© system identifies the maximum milliamperes per slice based on the region with the higher density then using the longitudinal and angular modulation. The software determines a reduction of the tube current relative for the low-density regions. The ATCM system is constantly updated and the machine adjusts the perceived image quality from the changes of the milliamperes per slice done by the NMT, who may override the milliamperes per slice suggested by the software (Wood et al. 2015).

The Siemens system (CARE Dose 4D ©) performs ATCM based on the user-defined image quality reference milliampere (QRM) value. The QRM value is selected according to the individual preferences of the user for the determined diagnostic requirements. The Siemens systems have stored referential images with the related QRM value for different X-ray attenuation models and different patient size, from the adult to the pediatric referential level. The values of minimum and maximum tube current ranges are also controlled and related to an ATCM adaptation software that can be set as low, average, or high. The default manufacturer settings provide an average decrease of the ATCM for slim, and an average increase for obese patients (Soderberg 2016).

For the Canon-Toshiba CT systems (SureExposure 3D ©), the image quality reference parameter is set by the desired standard deviation of pixel values in the reconstructed image, before the acquisition. The system modulates the tube current to achieve the stated value of standard deviation throughout the slices. The standard deviation can be set manually or using five default values to support the user. These predefined values range from “extra high quality” to “extra low dose” (Merzan et al. 2017).

The rotation time varies from 14 to 0.3 s, depending on the hybrid scanner class (Beyer et al. 2011a; Beyer et al. 2011b). Faster CT tube rotation time reduces acquisition time and therefore motion artifacts (moreover considering the wide range of rotation time of the hybrid scanner in nuclear medicine). The tube rotation is set on the basis of the clinical or technical cases. However, accelerating the rotation increases image noise and streak artifacts may occur but, at the same time, reduces patient’s effective dose, when all other parameters are kept constant.

In modern hybrid scanners, which have a multi-detector CT, the beam collimation represents a parameter strictly connected to the detector configuration, so it should be considered when the parameters are set. For example, a 16 × 0.625 would be 10 mm instead of the potential 20 mm. Therefore, to get a uniform flux over the detector array, the X-ray beam is slightly wider than the actual beam collimation, placing a penumbra region outside of the active detector arrays. This results in unused dose that does not contribute to image formation, also referred to as over-beaming (Raman et al. 2013b).

Increasing pitch during helical scanning offers the ability to reduce the time to scan a given volume with a resultant reduction in effective dose. However, high pitch reduces the density of the sampling of redundant data, introducing interpolation artifacts, increasing noise with a worsening of the signal to noise ratio (Primak et al. 2006) when all other parameters are kept constant and ATCM is disabled. It has been demonstrated that a pitch value of 1.4 can be an optimal compromise between image quality and speed of acquisition (Wang and Vannier 1997).

The NMT must be aware of the two acquisition modalities for cardiac gating: prospective and retrospective. In CCS imaging, the main modality is the prospective acquisition. This acquisition modality allows a decrease of effective dose up to 75% compared with retrospective gating (Morin et al. 2003; Hunold et al. 2003). Prospective cardiac gating is based on the estimate of a temporal phase of the R-R interval of the cardiac cycle. This specific, preselected time frame is identified before the scan itself by electrocardiogram (ECG) synchronization. The center of the acquisition window is located at approximately 70–75% of the R-R interval (Matsuura et al. 2008; Isma'eel et al. 2009).

Prospective ECG triggering is used in patients with low and stable heart rates (< 60 bpm). This technique assumes that the interval between two R waves remains stable for the entire duration of the acquisition (van der Werf et al. 2018). Modern CT scanners also provide the option to skip an acquisition in prospective ECG triggering mode if an extra systole occurs.

### Protocol standardization for CT-based attenuation correction

Nowadays, one of the most commonly used methods to manage the attenuation of the SPECT and PET photons in the body is CT-based attenuation correction (AC). The attenuation and scattering of photons in the body degrade image contrast, affecting quantification of activity and relative distribution of perfusion, resulting in a decrease or the image quality. CT-based attenuation correction is mandatory in cardiac PET, due to the substantial photon attenuation, while in SPECT, it is recommended, according to EANM guidelines (Verberne et al. 2015).

The CT-based attenuation correction provides a map of the attenuation coefficients based on the Hounsfield Unit (HU) of the CT scan. The HU are the values of relative attenuation using water and air as the reference values. The relationship between HU and the photon attenuation gives an opportunity to scale the measured attenuation at CT energies to that at the energies of SPECT and PET (Brown et al. 2008; Kinahan et al. 1998).

Each manufacturer has developed a protocol for AC and the manufacturer’s manual should be the first reference when new to the technique. However, there are some common CT parameters for the creation of CT-based attenuation maps, which are detailed in the next paragraph (Verberne et al. 2015).

The CT scans that are for attenuation correction only (with no intended diagnostic use) should be performed at the lowest possible settings. Typically, the tube voltage, depending on a manufacturer specification, range between the 70 and 140 kVp and it must be adjusted according to the patient size (Yu et al. 2010). Because it is normal to smooth the CT data prior to its use for attenuation correction, the noise has a minimal impact (Hulme and Kappadath 2014), so the image quality can be optimized/kept low to decrease the patient’s effective dose. Therefore, a low tube current (10–20 mA) together with a relatively high pitch, according to the manufacturer instructions, is recommended to decrease radiation exposure. The slice collimation and reconstructed slice thickness should be the approximate slice thickness of SPECT or PET (e.g., 4–7 mm) (Dorbala et al. 2013). Particular attention should be given to the gantry rotation speed. A slower rotation speed (at least 1/s or less) increases the effective dose; however, it is recommended in order to obtain a blur of the cardiac motion. This rotation rate allows the CT to sample the cardiac and respiration cycle in a similar fashion to the MPI where the image of the heart is averaged over several cardiac cycles during shallow tidal free-breathing (Dorbala et al. 2013). Misalignments can produce significant artifacts and errors in apparent uptake in the myocardial segments adjacent to lung tissue (Le Meunier et al. 2006)—particularly in PET. If a breath-hold protocol is used, the end-expiratory phase is recommended given that the non-breath-hold SPECT/PET data is mostly close to this respiratory phase (Dorbala et al. 2013). The field of view (FOV) of the protocol for AC should be the maximum diameter of the scanner, avoiding any truncation effect (Chan et al. 2016; Gregoriou et al. 1998).

Quality control of the registration between transmission and emission imaging is always required and software realignment must be performed to minimize remaining misalignment (Dilsizian et al. 2016).

Over the last decades, new reconstruction and correlation software algorithms and the use of picture archiving and communication system (PACS) have created the possibility to use a separate-external CT to generate an attenuation map. This can improve the quality of old generation cardiac CZT gamma camera that is without an integrated CT system (Agostini et al. 2016; Caobelli et al. 2016). In this kind of system, it has been recommended that AC, particularly where the evaluation of the inferior and inferolateral myocardium, is needed (Caobelli et al. 2016; Ito et al. 2017; Liu et al. 2015). Considering this, the NMT needs to be aware of the characteristics of the external CT used for generating the attenuation map.

In particular, the following critiria for the selection of the external CT must be taken into account:

- CT tube voltage validated to work with the attenuation correction algorithm for SPECT reconstruction (Verberne et al. 2015)
- Possibly non-gated images, preferring the average cardiac cycle CT images (Dorbala et al. 2013).
- CT and SPECT FOV should be compatible, meaning that the CT FOV should include the whole thorax. Since the used FOV in radiology could have a magnification applied and decreased pixel size that can exclude from the FOV part of the chest, depriving the attenuation map of the HU, due to the truncation effect (Chan et al. 2016; Gregoriou et al. 1998).
- Without any iodinated contrast agent (Bonta and Wahl 2010; Büther et al. 2007).
- Similar patient BMI between the SPECT acquisition and the previous CT acquisition (Thompson et al. 2005).
- Same arms position (over the head) (Prvulovich et al. 2000).
- Acquired without freezing a systolic or diastolic single cardiac cycle due to the fast tube rotation, causing potential misalignment between the CT and SPECT/PET (Dorbala et al. 2013).
- If no other CT system is available and the examination must be performed, a recent study (Fuchs et al. 2013) suggested the use of images obtained from spectral CT as feasible for AC. This technique, based on dual-energy technology, allows to remove the contrast agent, producing a virtual unenhanced image (Long and Fessler 2014).

### Coronary artery calcium scoring acquisition protocol

Coronary artery calcium is assessed by using non-contrast CT and it provides information about the amount of calcium deposits in the coronary arteries and therefore gives an indication of the risk factors for coronary events. It is independent from the risk factors associated with heart disease. Coronary calcium is related to the patient’s age and gender. The score that reflects the total area of calcium deposits and the density of the calcium is defined as the Agatston score and displays the risk percentile of a patient with coronary calcium (Agatston et al. 1990).

The CCS is a strong predictor of adverse cardiovascular events and it is important that technologists follow standardized protocols to ensure that the results are accurate. A high calcium score of an increased percentile would be an indication for further treatment, and it would be determined by the patient’s cardiologist (Greenland et al. 2007). The CCS could be preceded by a gated SPECT. When the calcium scoring is performed to complement the findings obtained from the gated SPECT, a major adverse cardiac event rate increases from an annual rate of 0.4% for patients without calcium in the coronary artery to an annual rate of ≥ 2% (similar to that of patients with established CAD) for patients with high Agatston scores (≥ 400) (Flotats et al. 2011).

Furthermore, the Agatston calcium score equal to zero is a useful information to identify the lowest risk class of patients (Greenland et al. 2004) that determines a reduction of the mortality risk in individuals at low to intermediate risk, considering a 15-year period (Valenti et al. 2015), and to exclude ischemic cardiomyopathy in patients with heart failure (Abunassar et al. 2011).

The manufacturer’s recommendations should be followed when performing CCS scan. Due to the differences between characteristics of the various CT systems, it is a challenging task to define acquisition protocols (Flotats et al. 2011).

Nevertheless, the following parameters represent a summary for a standard CCS scan (Detrano et al. 2005; Knez et al. 2002):

- CT images are acquired as ECG-triggered axial or sequential imaging, if possible avoiding the retrospective ECG gating, which increases the effective dose.
- Slice thickness analysis and reconstruction standardized to 2.5–3 mm and 1.5 mm slice increment to provide scores comparable to the CCS database.
- The milliamperes varying with patient body habitus and adjusted based on CT scout.
- The peak tube voltage fixed at 120 kVp to maintain the previously validated quantification by CT scan and the use of the 130 HU as standard threshold, voxels > 130 HU are defined as a calcified lesion.

During the post-processing, the CT reconstruction parameters used are crucial for the image quality. Since a 130-HU threshold is applied retrospectively on the direction of the main coronaries (left main artery, left anterior descending artery, left circumflex artery, and the right coronary artery) using specialized software, each factor that can affect the HU threshold should be taken into account, including the reconstruction parameters.

The detection threshold of drawing the regions of interest when detecting calcium deposits is 130 HU and the ranges of the density weighting factor are as follows:

- Factor 1: 130–199 HU
- Factor 2: 200–299 HU
- Factor 3: 300–399 HU
- Factor 4: > 400 HU

The Agatston score is derived by multiplying the calcification area (mm^2^) with the density weighting factor according to the maximum density (HU) of the calcification itself. Then the score of each region of interest from the main coronary arteries, regardless of the location, is summed, to determine the total Agatston score.

For example, a patient with a lesion area of 12 mm^2^ with an attenuation peak of 300 HU (factor 3) and a second calcification of 16 mm^2^, with an attenuation peak of 410 HU (factor 4), will have a total Agatston score equal to: [(12 × 3) + (16 × 4)] = 100. The Agatston score is evaluated by the clinician on the basis of the thresholds choice for the cardiovascular risk classification of the coronary calcium (Greenland et al. 2004; Detrano et al. 2008; Rumberger et al. 1999).

During the CCS post-processing every trans-axial slice needs to be scrutinized so that any calcification present in the coronary arteries is quantified. The NMT performing CCS needs to be aware of the patient’s history background, anatomy of the coronary arteries, including, their origin, direction, and location. The technologist might need to adjust the software processing since the 130 HU threshold makes no distinction between stents, noise, and calcium (Greenland et al. 2007).

Despite several prognostic data supporting the value of Agatston score for clinical risk predication as gold standard, all using the same reference protocol for CCS acquired at 120 kV and reconstructed at 3 mm slice thickness with filtered back projection (FBP), there have been changes in methodology, aiming to an optimization of the parameters (Blaha et al. 2017). For the CCS protocol optimization, the noise thresholds used in clinical practice of < 20 HU and < 23 HU are recommended for CCS imaging in small-/medium- and large-sized patients, respectively (Voros et al. 2011).

Iterative reconstruction (IR) has become the method of choice for processing CT scans (Willemink et al. 2013). IR algorithms improve image quality, by reducing noise and image artifacts compared to FBP. The noise reduction leads to a decrease of the calcium score due to a decrease in voxel intensity below the calcium threshold of 130 HU. This, in turn, improves the ability to detect smaller objects by eliminating the noise background (Takahashi et al. 2016; van Osch et al. 2014) and allows the decrease of the tube current with an effective dose reduction up to 75% with very low re-classification rates for cardiovascular risk stratification compared to FBP reference (Vonder et al. 2018).

Further relevant reconstruction parameters for CCS include the following: kernel (the filter to manage image noise, sharp, or soft, the choice of which depends on the tissue characteristics), slice thickness (that controls the spatial resolution in the longitudinal direction), reconstruction increment (the space or overlap between adjacent slices), and the display field of view (DFOV—determines how much of the object in the scan field of view is reconstructed into an image, similar to a zoom. If iterative reconstructions are used, in the raw data it is important that the full FOV is used—otherwise the comparison between forward projected data and observed do not agree). Since high image noise would increase coronary artery scores (van der Werf et al. 2017), reconstruction parameters are determinant for CCS measurement. Thinner slices thickness or increment and sharper kernels are associated with higher noise and mainly with upward re-classification of patient cardiovascular risk stratification. Higher DFOV causes minor changes in CCS assessment (Mantini et al. 2018). Therefore, soft kernels are recommended in CCS (Sprem et al. 2018), if thinner slices thickness and increment interval are applied. They should be compensated adapting the acquisition parameters and the reconstruction algorithms to obtain the recommended noise thresholds (Voros et al. 2011).

### Opportunities for combining AC and CCS

When establishing the benefits of the MPI combined with AC and CCS, several authors studied a dichotomous solution that optimizes the radiation exposure for the transmission imaging: using the AC to evaluate CCS (AC-CCS) or using the CCS to create a CT attenuation map (CCS-AC). These approaches led to removal of extra CT scan for the patient.

The efforts in the AC-CCS are all aimed at managing the limitations of the technique such as the difficulty of reliable CCS evaluation despite the cardiac motion in non-ECG-gated images or by patients’ non-compliance with breathing instructions, low image resolution, and high levels of image noise. On the other hand, the CCS-AC represents a challenging task due to the different acquisition method and the different anatomy compared to the AC where the slow tube rotation provides a systolic-diastolic mean of the heart HU.

The possibility to use the AC for CCS or the CCS for AC was explored by several authors. The examples described in this part are obtained through a literature search performed in December 2018 using Pubmed. The following search was used in Pubmed: ((coronar*) AND (calcium OR calcificat*) AND (scinti* OR myocardial perfusion or PET or SPECT) AND (attenuation or AC)). Inclusion criteria were as follows: studies published in the last 15 years; single or multicenter; either included phantom and ex vivo; description of the CT parameters; and emission and transmission imaging performed using a hybrid scanner, CCS-AC, or AC-CCS evaluation or optimization as purpose of the study. Exclusion criteria were as follows: non-English written full-text articles; abstracts without full text; editorials, reviews, case reports, and letters; and original studies using a slice thickness > 7 mm for visual analysis or Agatston score. If the CCS or AC were not performed using a hybrid scanner and instead used a stand-alone CT scanner, the study was not included in Tables 1 and 2, since both tables list only hybrid scanner parameters.

**Table 1:** Examples of hybrid system parameters of AC applied to evaluate CCS (AC-CCS). The not declared parameters are identified by the acronym (nd)

Authors	Einsteinn JA et al. (2010)			Engbers EM et al. (2016)	Mylonas I et al. (2012)	Išgum I et al. (2018)	Bailey G et al. (2017)
**Hybrid system**	SPECT/CT	PET/CT	PET/CT	SPECT/CT	PET/CT	PET/CT	SPECT/CT
**CT slices**	16	16	64	64	64	64	16
**ECG Gating - R-R interval (%)**	no	no	no	no	no	no	no
**Purpose**	AC- CCS	AC- CCS	AC- CCS	AC- CCS	AC- CCS	AC- CCS	AC- CCS
**Tube Potential / Current**	120 kVp / ∼50 mAs	120 kVp / ∼9 mAs	120 kVp / ∼11 mAs	120 kVp / 20 mA	140 kVp / 20 – 210 mA	100 kVp / ∼11 mAs	120 kVp / 30 mA
**Tube rotation (s)**	nd	nd	nd	0.8	nd	nd	0.4
**Pitch**	0.94	2	1.5	nd	0.984 : 1	1.5	0.81
**Slice thickness (mm)**	1.50	0.75	1.20	5.00 - 2.50	3.75	3.00	5.00
**CCS threshold (HU)**	visual analysis	nd	visual analysis	visual analysis	50 manual	130	130
**Breath**	free breathing	light end-expiratory breath hold	free breathing	end-expiratory breath-hold	mid-to-end expiration	end-expiratory breath-hold	nd

**Table 2:** Examples of hybrid system parameters of CCS applied to evaluate AC (CCS-AC). The not declared parameters are identified by the acronym (nd)

Authors	Burkhard N et al. (2010)		Ghafariana P et al. (2010)	Wenning E et al. (2013)	Zaidi H (2013)	Kaster TS et al. (2015)	Wu TH et al. (2015)
**Hybrid system**	PET/CT	PET/CT	PET/CT	SPECT/CT	PET/CT	PET/CT	PET/CT
**CT slices**	64	16	64	2	64	64	64
**ECG Gating - R-R interval (%)**	yes – RR nd	yes – RR nd	nd	Yes – 70	yes - RR nd	Yes – 70	Yes – 75
**Purpose**	CCS -AC	CCS -AC	CCS -AC	CCS -AC	CCS -AC	CCS -AC	CCS -AC
**Tube Potential / Current**	140 kVp / 400 mA	140 kVp / 400 mA	120 kVp / 190 mAs	130 kVp / 30 mAs	120 kVp / 190 mAs	120 kVp / 40 – 400 mA	120 kVp /obese: 50-150 mA; severely obese: 10-550 mA
**Tube rotation (s)**	0.35	0.5	0.33	0.57	0.33	nd	0.35
**Pitch**	nd	nd	0.2 : 1	nd	0.2 : 1	0.984 : 1	nd
**Slice thickness (mm)**	2.50	2.50	1.20	2.50	1.20	5.00	2.50
**CCS threshold (HU)**	nd	nd	nd	nd	nd	110	130
**Breath**	inspiration	inspiration	breath hold at end-inspiration	maximum inspiration	end-expiratory breath-hold	end-expiratory breath-hold	nd

One of the first attempts for the development of AC-CCS was by Einstein et al. (2010), who qualitatively compared the AC and the CCS scores from 492 patients from 3 centers including SPECT/CT and PET/CT patients. The non-gated AC was performed with free breathing or end-expiratory, while the calcium scoring scans were performed at expiration with ECG gating. The images acquired from both examinations were visually interpreted and analyzed. As a result, there was a high agreement between the visual calcium seen on the AC scans and the CCS scoring scans. A similar result was obtained also by Engbers et al. (2016). The group has demonstrated a high agreement of visual CCS from the AC with the Agatston CCS and excellent inter-reader reproducibility.

The NMT should take into consideration that the implemented institutional protocol for AC-CCS may use a different HU threshold compared to the value derived from the electron-beam CT-based protocol.

An example can be found in Mylonas et al. (2012). They performed a non-ECG-gated at the mid-to-end-expiration AC scan. Despite the higher tube voltage (140 kVp vs 120 kVp) and thicker slice (3.75 mm vs 2.5 mm), there was a high agreement between the CCS measured using cardiac CT and CCS using AC images from cardiac PET MPI. A manual threshold of 50 HU (instead of 130 HU) was used to identify the calcification. This was the first study to quantify CCS using AC images obtained during PET MPI in a cohort of 91 patients.

Išgum et al. (2018) moved back the threshold level to 130 HU because they stated that a 50 HU introduces more artifacts from the non-calcium structure for the set of images used in their study. They introduced a fully automatic calcium scoring system applied to AC images in clinical routine. The study included 128 patients who underwent the PET MPI study with an AC and a consecutive CCS scan. Both CT scans were assessed by a manual and an automatic calcium scoring system and then compared. In addition, the cardiovascular disease risk categorization from CCS was investigated. The results of the study demonstrated that an automatic CCS scoring from AC combined with a fast-visual quality control of the calcific region may allow routine CAD risk assessment. The same threshold was applied by Bailey et al. (2017). They identified 40 patients in an elevated risk population who underwent lung cancer screening computed tomography (non-gated) (LCS-CT), CCS, and AC-CT within the same 2-year period. Despite LCS-CT and AC-CT had a comparable area under the receiver operating characteristic curve (ROC), the LCS-CT, in comparison with AC-CT, had a better correlation with the calcium score values and a comparable risk assessment to the CCS CT scan. Moreover, a recent guideline by Society of Cardiovascular Computed Tomography and the Society of Thoracic Radiology (SCCT/STR) supports the non-gated CT as a useful tool to report moderate or severe coronary artery calcification (Hecht et al. 2017). A summary of the parameters used in the AC-CCS is available at the Table 1.

The use of the CCS for attenuation map was investigated both for PET and SPECT. The effect on quantitative and qualitative PET MPI reconstructed using a CCS-AC was analyzed. One of the first attempts to evaluate the use of the CCS as AC was done by Schepis et al. (2007) importing an external CT performed for CCS. This study demonstrated for the first time that the use of CT data from CCS obtained with a 64-slice CT allowed accurate AC of myocardial SPECT MPI images. The attenuation corrected SPECT images obtained using multi-detector CT data from CCS (either AC during inspiration phase or AC in expiration) resulted in identical qualitative and semi-quantitative data compared with the standard AC reconstruction of the SPECT MPI.

Burkhard et al. (2010), acquiring both CT and emission data on a PET/CT hybrid system, assessed the values of myocardial blood flow (MBF) and coronary flow reserve (CFR) of a PET MPI using an ECG-triggered CCS scan for AC. MBF values were calculated using AC maps obtained from the CCS scan during inspiration and validated against MBF values calculated using standard AC. The CCS scan provided results highly comparable to those obtained with conventional AC. There was an excellent correlation between AC and CCS for segmental and global MBF values at stress-rest as well as for CFR.

Ghafarian et al. (2010), using ^18^F-FDG and ^13^N-NH_3_ PET/CT, reported no significant differences between the CCS-AC and the AC. These results are in a line with the report by Zaidi et al. (2013). The semi-quantitative analysis by perfusion defect scores and the quantitative by MBF and CFR were also performed and seemed to be reliable. This study demonstrated that an improvement could be obtained using a CCS-AC at the end-expiration for attenuation correction of cardiac perfusion PET studies to quantify relative perfusion and MBF; this allowed to perform the CCS-AC in the same respiratory phase, as recommended by guideline for AC, when a breath-hold protocol is applied.

Wenning et al. (2013) investigated the CCS-AC in SPECT. Despite the limitation of a two-sliced CT scanner, qualitative image analysis and interpretation showed similar results for both types of CT scan and the image interpretation includes uncorrected images as well. The semi-quantitative evaluation of perfusion defect scores showed a significantly higher frequency and an enhanced severity of defects when CCS was used as AC. Probably, as stated by the author, an institutional CCS-AC normal database may improve the quantitative evaluation. In this study, it was noted that a large misalignment between CT and NM emission part can occur, requiring a manual alignment.

Kaster et al. (2015) have studied the optimization of the CCS-AC to determine if a low-dose scan can quantify coronary artery calcium and provide an AC map. A cohort of 23 patients underwent both a traditional CCS-CT and a PET/CT MPI. A modified low-dose CCS-AC was performed. Lowering the calcification threshold from 130 to 110 HU, they obtained an excellent agreement between the CCS-AC (measured at 110 HU, using 5 mm slices) and standard CCS-CT calcium scores (*r*^2^ = 0.99).

The effective dose for this modified CCS-AC was ~ 0.5 mSv. In addition, the authors suggested performing CCS-AC scan under resting conditions due to a lower heart rate. This study introduced the possibility to use a single low-dose CT scan to perform AC in rest and stress PET MPI and allowing a second low-dose CT to improve the quantification of coronary artery calcium using optimized parameters. Wu et al. (2015), using an anthropomorphic cardiac phantoms simulating normal weight, mildly obese, and severely obese patients, verified the feasibility of low-dose CT protocols for CCS and PET attenuation correction in cardiac PET/CT to assess patients in different weight categories. The parameters used in these studies about CCS-AC are shown in Table 2.

A further optimization of the CCS-AC can be found in the study by Grani et al. (2018). This study has focused on the effective dose reduction by decreasing the tube peak voltage. The group, using a newest generation 256-slice CT scanner, has recently shown that calculation of CCS from ultra-low-dose CT scans may be accurate if adjusted thresholds are applied. The first threshold proposed was moved to 145 HU using 100 kVp, 177 HU if 80 kVp is applied and 207 for the 70 kVp, reducing mean effective dose per patient from 0.60 mSv to 0.19 mSv and 0.12 mSv respectively. The data from this previous study was used also by Grossmann et al. (2018) to evaluate and develop an attenuation map. The group has validated the previous CCS ultra-low-dose CT in comparison with the reference standard tube potential (120 kVp) for AC in MPI, obtaining accurate and comparable results of the attenuation maps between the 70–80 kVp and the 120 kVp-CT.

It is important to underline that the classification of the calcium scores derived from AC-CCS or CCS-AC was in agreement with the CCS-CT in the classification of risk categories. The AC-CCS non-gated protocol could be developed to identify moderate or severe coronary artery calcification (Hecht et al. 2017). So independently of AC-CCS or CCS-AC, according to the institutional protocol chosen, the NMT must be aware of the bias factors and influencing the respective artifacts.

If an AC-CCS protocol is chosen for a clinical setting the NMT must take into account that if a different voltage is used, the HU threshold level must be adjusted according to the used tube voltage, and the threshold must be reduced if a voltage is set greater than the standard 120 kV or increased in the opposite case. The study by Grani et al. (2018) identified the thresholds if the tube voltage is reduced. If the study is applied to the AC-CCS images instead of CCS images, it is likely these thresholds must be adjusted and increased due to the blurring in the non-gated images which leads to an underestimation of the CCS. Further studies are therefore required to identify the AC-CCS thresholds when the tube potential is lower than 120 kVp. When greater tube voltage values are applied, CCS evaluation needs to be supported by a manual analysis (Mylonas et al. 2012). A low tube current can increase the image noise increasing consequently the CCS. Larger slices thickness than the standard (3 mm) lead to significantly reduced scoring results with the opposite found using thinner slices (Christensen et al. 2019) where soft kernels are recommended in CCS (Sprem et al. 2018). Looking at the outperformance of the LCS-CT in the study by Bailey et al. (2017), despite the intrinsic limitation, so long as appropriate an acquisition has been made it can be reasonable having two reconstructions from a single AC-CCS study, one for the AC, using 5 mm thickness and the full SFOV, and one for CCS, using a 2.5–3 mm slice thickness and a maximum DFOV of 400 mm, as used for LCS-CT.

The CCS-AC gated protocol, since the image is not averaged and blurred as in the emission part, could affect the emission image quality, semi-quantification, and absolute quantification. Therefore, the gated protocol requires manual alignment of images. Furthermore, the development of institutional CCS-AC 'normal' database will improve the MPI assessment.

Although both protocols require more investigations to optimize the technical parameters, both AC-CCS and CCS-AC can be a useful resource for the classification in risk categories of the patients, so the NMT must know how to manage the base of each one to contribute to the development of the techniques.

## Conclusions

Nuclear cardiology is still developing within nuclear medicine applications. Specific knowledge, skills, and competencies about hybrid nuclear cardiology are mandatory for completing NMT education system. Understanding each patient’s characteristics and conditions allows the NMT to adjust the protocol, bringing significant benefits for every patient. Additionally, a careful and knowledge-based choice of CT parameters results in a reduction of the effective dose for the patient, while maintaining an acceptable image quality.

Therefore, it is essential for NMT’s education and training to have full knowledge of the CT acquisition parameters and protocols, as well as an understanding of the differences among CT scanners, to ensure that the patient gets the best examination in terms of both diagnostic quality and effective dose. This is a hallmark for best practice. A specific education and training for NMTs and a cooperation with CT manufacturer specialists can help to achieve these purposes.

## Data Availability

Not applicable
